# Review of Electroanalytical-Based Approaches for the Determination of Benzodiazepines

**DOI:** 10.3390/bios9040130

**Published:** 2019-11-02

**Authors:** Kevin C. Honeychurch

**Affiliations:** Centre for Research in Biosciences, Department of Applied Sciences, University of the West of England, Frenchay Campus, Bristol BS16 1QY, UK; kevin.honeychurch@uwe.ac.uk

**Keywords:** benzodiazepine, electrochemistry, voltammetry, polarography, biomedical, forensic, analysis, review

## Abstract

The benzodiazepine class of drugs are characterised by a readily electrochemically reducible azomethine group. A number are also substituted by other electrochemically active nitro, N-oxide, and carbonyl groups, making them readily accessible to electrochemical determination. Techniques such as polarography, voltammetry, and potentiometry have been employed for pharmaceutical and biomedical samples, requiring little sample preparation. This review describes current developments in the design and applications of electrochemical-based approaches for the determination of the benzodiazepine class of drugs form their introduction in the early 1960s to 2019. Throughout this period, state-of-the-art electroanalytical techniques have been reported for their determination. Polarography was first employed focused on mechanistic investigations. Subsequent studies showed the adsorption of many the benzodiazepines at Hg electrodes allowed for the highly sensitive technique of adsorptive stripping voltammetry to be employed. The development and introduction of other working electrode materials such as carbon led to techniques such as voltammetry to become commonly reported, and the modification of these electrodes has now become the most commonly employed approach using molecularly imprinting and nanotechnology.

## 1. Introduction

Since the introduction of chlordiazepoxide hydrochloride (Librium^®^) in 1960 [1], a large number of structurally similar 1,4- and 1,5-benzodiazepine compounds have been developed and have been utilised widely as tranquillisers, hypnotics, sedatives, and antidepressants [2], and are presently one of the most commonly prescribed class of drugs. They were originally developed by Dr. Leo Sternbach in his doctoral degree studies at Jagiellonian University in Krakow, and then further explored during his employment in New Jersey, in the USA, with Wallace Pharmaceuticals. The company had already developed a GABA receptor-binding compound, meprobamate, which demonstrated powerful tranquillising and sedative effects, but which also exhibited dependence and abuse potential. Charged with finding a more benign alternative, Sternbach turned to his previous doctorial research on benzodiazepines. After exploring, unsuccessfully, over 40 different compounds over a period of two years, in 1956, animal testing of a material he named Ro 5–0690 showed definite tranquillising effects with no apparent side effects. This compound was later named methaminodiazepoxide, and then chlordiazepoxide (Librium^®^), and was approved for use in 1960. Librium^®^ was then followed in 1963 by diazepam (Valium^®^), and today, there are more than 1000 benzodiazepines synthesised [3]. Presently, this group of compounds represents one of the most commonly prescribed drugs. Over the period 2009 to 2014, the number of prescriptions dispensed for benzodiazepines in the USA increased by 226% to 133.4 million [4].

Compared to older sedatives, the application of benzodiazepines was considered to result in less side effects, such as toxicity, abuse, physical dependence, and suicide potential. They also exhibit a much improved therapeutic ratio, of above 100 compared to only 10 for the older drug classes, such as barbiturates [5]. However, more recent studies have shown a growing controversy focused on their usage [6]. Prolonged usage has been found to result in increased tolerance, physical dependence, increased aggression, and withdrawal symptoms. Overdose can result in symptoms like respiratory depression or coma. Reviews [7,8] have highlighted issues with their use with groups, particularly the elderly, with increased reports of falls, incidence of hip fractures, and risk of cerebrovascular events and deaths.

Due to their wide pharmaceutical usage and subsequent disposal, concern has also focused on other areas affecting the wider population. Reports have highlighted their use in drug-facilitated crime (DFC), drug-facilitated sexual assaults (DFSA) [9,10,11], and their adverse effects on activities such as driving [12,13]. Detectable levels have also been recently reported in potable and environmental water systems, resulting in possible toxic effects for both humans and aquatic life [14,15,16,17]. Consequently, there is a pressing demand for methods capable of determining trace levels of benzodiazepine in a variety of samples, including both environmental and medical. Common analytical approaches utilised [18,19,20] for the determination of benzodiazepines, such as gas chromatography and immunoassay [21] and liquid chromatography [22], have recently been reviewed. However, to our knowledge, the application of electrochemical-based techniques for their determination has not been recently reviewed.

## 2. Electrochemical Behaviour of Benzodiazepines

Benzodiazepines are characterised by a relatively easily reducible azomethine group with a number also containing other electrochemically active groups, including nitro, *N*-oxide and carbonyl groups (Appendix A). As a result, many common electrochemical techniques, such as polarography, voltammetry, and also potentiometry, have been successfully utilised for their determination. Electrochemical-based approaches offer advantages in sensitivity, economy, and flexibility, and have been utilised for the successful determination of a range of pharmaceuticals and their metabolites in complicated matrices such as blood and urine [23,24,25], and for forensic analysis [26,27,28,29]. Electrochemistry has also been shown suitable for analytical quality control of pharmaceutical formulations, utilised for quantitative determinations and studies of impurities, and it has also been used in biological and mechanistic studies. Utilising techniques such as adsorptive stripping voltammetry (AdSV) detection limits in the pM range have been reported (Table 1).

This review focuses on electrochemically-based approaches for their determination. The subject is divided into two main areas, polarographic and voltammetric applications, including details of methodology, design, and performance of selected applications. Those utilising liquid chromatography with electrochemical detection have been reviewed recently [30]. Discussion is focused on research papers published in the past 12 years. Reports made prior to 2006 are summarized in Table 1, Table 2 and Table 3. A number of these earlier investigations have already been reviewed [23,31,32,33,34,35,36,37].

## 3. Mercury-Based Working Electrodes

Mercury has been proven to be an extremely good electrode material and has been used for a wide number of applications for a considerable time [38], with reports on the polarographic behaviour of chlordiazepoxide hydrochloride (Librium^®^) being made as early as 1963 by Oelschläger [39]. The polarographic determination of benzodiazepines offers a number of advantages, as these drugs are readily adsorbed at the Hg electrode surface, allowing for the highly sensitive technique of AdSV to be employed.

More recently, tetrazepam has been determined at a Hg drop electrode utilising Britton-Robinson buffer modified with 10% ethanol as the supporting electrolyte [40]. Several electrochemical approaches were investigated, including differential pulse polarography (DDP), differential pulse cathodic adsorptive stripping voltammetry (DPAdCSV), linear sweep cathodic adsorptive stripping voltammetry (LSAdCSV), and square wave cathodic adsorptive stripping voltammetry (SWAdCSV), gaining detection limits of 5 × 10^−6^, 3 × 10^−7^, 1 × 10^−8^, and 3 × 10^−9^ M, respectively. Bulk, pharmaceutical formulation, and human serum were investigated, and the adsorptive stripping voltammetry procedures developed were shown to be able to successfully determine tetrazepam in human serum without any prior pre-treatment.

Reports have shown that pharmaceuticals such as benzodiazepines are increasingly used to illegally fortify complementary and alternative medicines [28]. Clonazepam, flurazepam, alprazolam, midazolam, medazepam, chlordiazepoxide, and diazepam were successfully determined in phytotherapeutic formulations at a Hg drop electrode, gaining a linear range of 1.0 × 10^−6^ to 30.0 × 10^−6^ M, utilising a pH 10 Ringer buffer solution as the supporting electrolyte. The benzodiazepines determined were divided into four separate groups according to their cathodic voltammetric behaviour, permitting the rapid voltammetric scanning of these formulations.

The voltammetric behaviour of nitrazepam has been recently reported at dropping Hg and glassy carbon electrodes [41]. Voltammetric investigations utilising a pH 5.7 acetate buffer containing 1% sodium lauryl sulphate showed two well-defined reduction peaks resulting from the 4e^−^, 4H^+^ reduction of the 7-nitro group to the corresponding hydroxylamine and the 2e^−^, 2H^+^ reduction of the azomethine group. A detection limit of 0.47 × 10^−7^ M was reported, and the assay was successfully employed for the determination of nitrazepam in tablet formulations.

The determination of nordiazepam has been studied at the Ag–Hg amalgam electrode [42]. Utilising DPV with a supporting electrolyte of a pH 10 Britton-Robinson buffer and methanol (9:1), a linear range from 2 × 10^−6^ to 1 × 10^−4^ M with a corresponding limit a quantification of 1.7 × 10^−6^ M was reported. The Ag–Hg amalgam electrode was fabricated by immersing an Ag electrode in liquid Hg for 15 s. This was then electrochemically activated at a potential of −2.2 V in 0.2 M KCl. The electrode was periodically regenerated by switching every 0.1 s between the potentials −1.4 V and −0.8 V.

Latterly, Nunes et al. [16] have utilised differential pulse cathodic adsorptive stripping voltammetry to determine the occurrence of alprazolam in Brazilian river water to evaluate its potential use as a marker of contamination by wastewater. Investigations showed that alprazolam was reduced at a HMDE giving a single irreversible reduction peak at −0.95 V in pH 7 phosphate buffer concluded to result from the 2e^−^, 2H^+^ reduction of the azomethine group. Peak current was found to increase proportionally with scan rate, demonstrating the electrochemical mechanism to be adsorptive in nature. Conditions were optimised for the determination of alprazolam in environmental water samples, and a limit of quantification of 0.4 μg/L for a 120 s pre-concentration time was reported with recoveries ranging from 93% to 120%. Analyses of water samples taken downstream of a sewage treatment works on the Cascavel River were found to have an alprazolam level of 5.9 ± 0.5 µg/L.

As can be seen form Table 1, a large percentage of previous reports have utilised Hg-based electrodes. However, the use of Hg has fallen from favour due to problems with toxicity and disposability. Consequently, a number of recent reports have investigated alternative approaches, which are illustrated in more detail in the next section.

## 4. Mercury Free Electrodes

### 4.1. Metal Electrodes

Channaa and Surmann [97] utilised a galinstan drop electrode as an alternative to Hg for the determination of chlordiazepoxide, nitrazepam, and diazepam. Galinstan is an alloy of gallium, indium, and tin, which is a liquid at room temperature, making it a good substitute for the potentially more toxic Hg. Differential pulse and adsorptive stripping voltammetry at this electrode showed very similar behaviour to that observed at Hg drop electrodes. In another investigation, Tyszczuk [98] utilised an in situ plated lead film glassy carbon electrode for the determination of temazepam and diazepam in tablet formulations and oxazepam in human urine. Using an electrolyte of pH 4.6 0.05 M acetate buffer containing 5 × 10^−5^ M Pb, detection limits of 2.0 × 10^−9^, 20 × 10^−9^, and 5.0 × 10^−9^ M for diazepam, temazepam, and oxazepam, respectively, were obtained.

Square wave voltammetry has been utilised to determine the adsorption of lorazepam at a gold ultramicroelectrode (25 µm diameter) in a 0.05 M H_3_PO_4_ [99]. A linear response was found over the range 10^−11^ to 10^−6^ M, with a corresponding detection limit of 6 × 10^−12^ M (ca. 2 pg/mL). The method was based on measuring changes in the admittance of a gold ultramicroelectrode (in 0.05 M H_3_PO_4_ solution) caused by adsorption of the lorazepam of the electrode surface. The signal-to-noise ratio was significantly improved by application of the discrete fast Fourier transform method, background subtraction, and two-dimensional integration of the electrode response over a selected potential range and time window. The method was evaluated by determining the lorazepam content of commercial available tablet formulations.

Doménech-Carbó et al. [100,101] investigated the adulteration of phytotherapeutic formulations with a number of different drugs, including the benzodiazepines: Clonazepam, flurazepam, alprazolam, midazolam, bromazepam, chlordiazepoxide, lorazepam, and diazepam. Paraffin-impregnated graphite electrodes (PIGE) were gently rubbed in a suitable portion of the powdered pharmaceutical formulation under investigation. This was then rinsed with water and transferred to the electrochemical cell containing pH 4.75 acetate buffer as the supporting electrolyte. The physically adsorbed film on the electrode surface was then investigated by square wave voltammetry. The presence of benzodiazepine within the various formulations could then be identified by their different reduction peak potentials. In further studies, quantification was also shown to be possible using a modified multiple standard addition method [101].

### 4.2. Carbon Electrodes

El-Sayed et al. [102] studied the electrochemical behaviour of chlordiazepoxide at both a glassy carbon electrode (GCE) and at a mercury drop electrode. Under acidic conditions, three irreversible reduction waves were reported, corresponding to three separate twoelectron reduction steps. Cyclic voltammetric investigations over the potential range 0.0 to −1.2 V showed two reduction peaks, with no oxidation peaks seen on the return positive going scan. The cathodic adsorptive stripping voltammetric behaviour of chlordiazepoxide was studied at the GCE. Using a linear-sweep waveform for quantitative determination of chlordiazepoxide in serum and commercial tablets by dilution in pH 4 Britton-Robinson buffer, a linear relationship between peak current and drug concentration was obtained over the range 2 × 10^−7^ to 5 × 10^−6^ M, with a corresponding detection limit of 5 × 10^−8^ M. Utilising a relatively simple sample preparation via dilution with buffer, recoveries ranging between 97 and 102.5% were reported for both sample types.

Recently [103], the voltammetric behaviour of bromazepam and alprazolam were investigated at both laboratory-fabricated and commercially available unmodified and Hg-free boron-doped diamond electrodes (BDDE). Cyclic voltammetric investigations were reported to show that both bromazepam and alprazolam underwent an irreversible and diffusion-controlled reduction process at these electrodes. Bromazepam was reported to give a single reduction peak at −1.10 V in a pH 11 Britton-Robinson buffer for both the commercial and laboratory-fabricated electrodes. Alprazolam was reported to give a single peak, with a peak potential of −0.84 V in a pH 5 Britton-Robinson buffer of pH 5 at the commercial BDDE. Using differential pulse voltammetry, linear calibration curves were obtained for bromazepam and alprazolam from 1 × 10^−6^ to 1 × 10^−4^ and 8 × 10^−7^ to 1 × 10^−4^ M, with detection limits of 3.1 × 10^−7^ and 6.4 × 10^−7^ M, respectively. Using the optimised conditions, investigations of pharmaceutical tablets were reported to give mean recoveries of 97.3% to 100.9% for bromazepam and from 94.1% to 101.3% for alprazolam.

The five benzodiazepines—clonazepam, diazepam, alprazolam, chlordiazepoxide, and oxazepam—have been determined at a poly dopamine-poly folic acid (PDA–FA) nanocomposite-modified GCE [104]. Investigations using cyclic voltammetry were reported to show that the modified GCE exhibited enhanced voltammetric responses for the oxidation of these five benzodiazepines in alkaline solutions. The authors investigated the possibility of determining the benzodiazepines by DPV. The method was successfully applied for the determination of the drugs in human plasma samples. Recoveries were reported to range between 92.5% and 110%. Following polishing with 0.05 μm alumina, a dopamine/folic acid film was electrochemically polymerised on the GCE surface. This was achieved by repeated cyclic voltammetric scanning over the range −1.0 to +1.0 V, at a scan rate of 100 mV/s in a 0.1 M phosphate saline pH 7.4 buffer containing 0.5 mg/mL dopamine. Once 15 cyclic voltammetric cycles were completed, the dopamine peak currents were reported to become constant. The modified GCE was then transferred to a separate 1 mM HCl solution containing 0.9 mM folic acid, and repeated cyclic voltammetric scanning over the potential range −1.0 to +1.5 V at a scan rate of 50 mV/s for 10 cycles.

### 4.3. Graphene-Modified Electrodes

Chlordiazepoxide has also been investigated by Motaharian and Hosseini [105] at a grapheme-carbon paste electrode (GCPE) modified with imprinted polymer nanoparticles (nano-MIP). Chlordiazepoxide imprinted polymer nanoparticles were prepared by a suspension polymerisation method from methacrylic acid (functional monomer), ethylene glycol dimethacrylate (cross-linker), and 2,2-azobisisobutyronitrile (initiator) in acetonitrile. This pre-polymerisation mixture was added to the 60 mL of nitrogen-purged silicon oil and dispersed by stirring and ultrasonication. After heating to complete polymerisation, the polymer particles were filtered and washed with petroleum ether and toluene. The chlordiazepoxide and un-polymerised components were then removed by washing with methanol/acetic acid and ethanol and dried. Modified carbon paste electrodes were prepared from graphite powder, graphene, MIP modifier, and n-eicosane. This was then packed into the end of a glass holder and an electrical connection made with a copper wire. The modified electrode was incubated in a CDP solution (pH 3.5) for 10 min with stirring, and then washed and placed in an electrochemical cell containing 10 mL of a 0.1 M sulphuric acid solution, and the concentration of chlordiazepoxide determined by square wave voltammetry. The nano-MIP-modified electrodes exhibited much greater adsorption towards chlordiazepoxide than the non-imprinted polymer nanoparticle. They also showed that the inclusion of graphene in the preparation of the carbon paste electrode gave a significant improvement in response. A linear range from 6.0 × 10^−10^ to 7.5 × 10^−8^ M (*R*^2^ = 0.9982) was reported with an associated detection limit of 2.61 × 10^−10^ M. The determination of chlordiazepoxide in pharmaceutical formulations, human serum, and urine samples was shown possible, with mean recoveries of 99.0%, 94.5%, and 97.8%, respectively, reported.

Rezaei et al. [106] determined lorazepam at a pencil graphite electrode (PGE) modified with an electro-polymerised molecularly imprinted polymer (MIP) formed from polypyrrole (ppy), sol-gel modified with Au nanoparticles (AuNPs). The surface of the PGE was first pre-treated by applying a constant potential of +1.40 V for 300 s in a 0.5 M pH 4.8 acetate buffer supporting electrolyte, containing 0.02 M NaCl. The pre-treated electrode was immersed in a sol formed from tetraethoxysilane (TEOS), phenyltriethoxysilane (PTEOS), trifluoroacetic acid (TFA), ethanol, lorazepam, pyrrole, sodium dodecyl sulfate (SDS), and hydrogen tetrachloroaurate trihydrate (HAuCl_4_ 3H_2_O). The potential of the PGE was then scanned over the range of −0.80 to +0.60 V (vs. Ag/AgCl) by cyclic voltammetry (50 mV/s for 7 cycles) to give a polypyrrole@sol-gel@Au nanoparticle MIP film. Lorazepam was extracted from the MIP film by immersion in a methanol-acetic acid (50% *v*/*v*) solution. The fabrication process of the sensor was characterised by cyclic voltammetry and electrochemical impedance spectroscopy (EIS). Under the optimised conditions, linear responses from 0.2 to 2.0 nM and 2.0 to 20.0 nM were reported, with a detection limit of 0.09 nM. The possibility of utilising the developed sensor for the determination of lorazepam in tablet formulations, plasma, and urine was investigated and mean recoveries of 102% were reported for all three sample types. Panahia et al. [107] utilised the alternative precipitation polymerisation method to modify the surface of a carbon paste electrode with a MIP for the determination of midazolam. Linear responses of 5.0 × 10^−10^ to 1.0 × 10^−7^ M and 1.0 × 10^−7^ to 1.0 × 10^−6^ M were reported, with corresponding detection limits of 1.77 × 10^−10^ M. The sensor was successfully used to determine midazolam concentrations in pharmaceutical formulations and human urine, gaining reported mean recoveries of 101.5% and 93.1%, respectively.

Ag–Pt core–shell nanoparticles supported on graphene nano-sheets (Ag–Pt/GRs/GCE) have been used to fabricate a sensor for oxazepam [108]. These were shown to be able to successfully determine of oxazepam in serum, urine, and pharmaceutical preparations. Voltammetric investigations showed a reportedly significant enhancement of the reduction current, and also a reduction in overvoltage required. Ag–Pt/GRs were prepared by mixing H_3_PO_4_/H_2_SO_4_ and graphite/KMnO_4_ at 60 °C 15 h before the addition of 30% H_2_O_2_. Following centrifugation, and filtration, the graphene oxide was washed with water, 30% HCl, and finally ethanol. The GO powder was ultrasonically dispersed in aqueous AgNO_3_ stabilised with sodium citrate solution. After the addition of sodium borohydride aqueous solution, the product was collected by centrifugation and dispersed into an ethylene glycol/water and finally filtrated, washing with water and dried. The GCE was then modified by drop-coating the silver–platinum core–shell nanoparticles supported and graphene and drying at room temperature. The electrochemical parameters were optimised, and a linear range of 0.05–150.0 μM (*R* = 0.9997) and a detection limit of 42 ± 1 nM for oxazepam by differential pulse voltammetry was reported. Analysis of oxazepam over the concentration range 5 to 120 µM in urine and human blood serum gave reported recoveries of 99.9% and 102%, respectively. Using the developed device, oxazepam tablets were reported to contain 94 mg/tablet, within 6% of the stated value.

### 4.4. Nanoparticles, Carbon Nanotubes, and Fullerene-Modified Electrodes

The reported synergic effect of Au nanoparticles and graphene has been investigated for the possible voltammetric determination of nitrazepam [109]. Using differential pulse voltammetry, a linear range of 0.5 to 400 μM with a corresponding detection limit of 0.166 μM was reported. Recoveries of between 99% to 102.4% were reported for serum and a pharmaceutical formulation. The modified electrode was fabricated by first drop casting an aqueous suspension of reduced graphene oxide onto a GCE. This was then immersed in an acetate buffer solution containing 1 mM HAuCl_4_. Au nanoparticles were then deposited from this solution onto the modified GCE surface via repeated cyclic voltammetric scanning over the potential range −0.2 V to +1.2 V (vs. Ag/AgCl), at a scan rate of 100 mV/s. The Au nanoparticle/graphene nanostructure was characterised by Fourier transform infrared spectroscopy, atomic force microscopy, scanning electron microscopy coupled with energy dispersive X-ray spectroscopy, and voltammetry.

Recently, Rahimi-Nasrabadi et al. [110] explored the possibility of determining diazepam in serum, urine, and tablets at a fullerene-functionalised carbon nanotubes/ionic liquid nanocomposite-modified GCE. The electrode was modified using fullerene-functionalised carbon nanotubes and ionic liquid (1-butyl-3-methylimidazolium tetrafluoroborate). The properties of fullerene-functionalised carbon nanotubes and ionic liquid were characterised by transmission electron microscopy, scanning electron microscope, electrochemical impedance spectroscopy, and voltammetry. It was reported that the modified GCE showed electrocatalytic activity toward the reduction of diazepam and a linear response over the range 0.3–700 µM, with an associated detection limit of 87 ± 2 nM being obtainable. Mean recoveries of 100% and 101% were reported for urine and serum, respectively.

The possibility of determining clonazepam has been investigated at a poly melamine and multiwalled carbon nanotubes nanocomposite (PMela/CNTs)-modified GCE [111]. The GCE was modified by first drop casting an aqueous suspension of MWCNTs on to its surface. An electrochemically-polymerised layer of polymelamine was then added by cyclic voltammetric scanning over the potential range +0.1 to +1.8 V for 15 cycles at a scan rate of 100 mV/s in a 0.1M HCl solution containing 1.0 mM melamine. Following a 100 s accumulation time, a weak and broad peak was observed at −0.72 V at the unmodified GCE surface, whereas a well-defined and distinct cathodic peak was reported for clonazepam at −0.58 V at the PMela/CNTs/GCE. The peak current for this peak was reported to be ca. 15 times greater than that reported for the peak current at the bare GCE. This increased adsorption was reported to be a result of the interaction between clonazepam and polymelamine/MWCNTs and the π–π interaction between the aromatic ring of clonazepam and the structure and the benzene rings of MWCNTs. Employing the optimised conditions, a linear square wave voltammetric response was reported over the range 0.05 to 10 µM, with a detection limit of 63 nM (3 σ).

Ashrafia et al. [112] have reported the development of an ink based on Ag nanoparticle–nitrogen-doped graphene quantum dots (Ag/N-GQD) for the determination of alprazolam, chlordiazepoxide, diazepam, oxazepam, and clonazepam in pharmaceutical and human plasma samples. The polymeric conductive film was electrodeposited on the surface of the poly chitosan-modified Au electrode. Both DPV and SWV were explored for the determination of the five benzodiazepines. By DPV, the limit of quantification (LOQ) for alprazolam and chlordiazepoxide was reported as 56 µM and 52 µM, respectively, compared to 73 µM and 70.4 µM by SWV. However, DPV was less sensitive for clonazepam with a LOQ of 84 µM compared to 54 µM by SWV. Using a 0.1 M NaOH supporting electrolyte, it was reported possible to determine the drugs in diluted human plasma.

### 4.5. Screen-Printed Electrodes

**Scheme 1:**
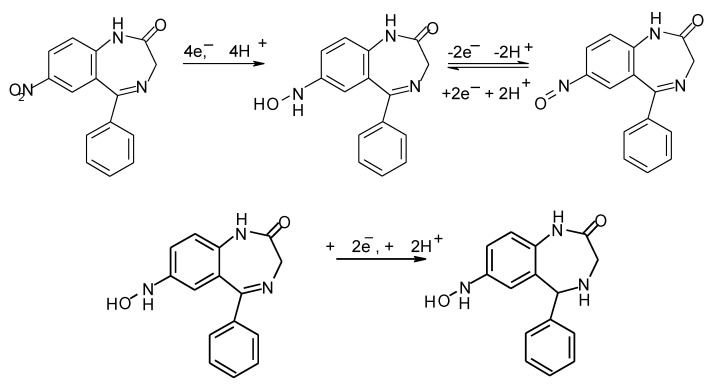
Proposed voltammetric redox behavior of nitrazepam at a screen-printed carbon electrode.

As can be seen from the preceding section, the majority of voltammetric assays utilise the 2e^−^, 2H^+^ reduction of the 4,5-azomethine bond; however, in the case of species such as nitrazepam, the reduction of the nitro group can be utilised as the analytical signal [34,35,103,113]. However, these approaches can suffer from some certain drawbacks, as the simultaneous reduction of oxygen present in the sample can result in interfering reduction waves. McGuire et al. [114] recently utilised an alternative approach for the adsorptive anodic stripping voltammetric determination of nitrazepam in beverage samples at a screen-printed carbon electrode (SPCE). Cyclic voltammetric investigations showed the 7-nitro group to be reduced to the corresponding hydroxylamine (Scheme 1). A second reduction peak was recorded at more negative potentials resulting from the 2e^−^, 2H^+^ reduction of the 4,5-azomethine group. Once generated, the hydroxylamine can be re-oxidised to the nitroso species at potential close to 0 volts. The use of this oxidation process as the analytical signal has several advantages, as many common electrochemical interferences are not seen at such low applied potentials.

**Figure 1:**
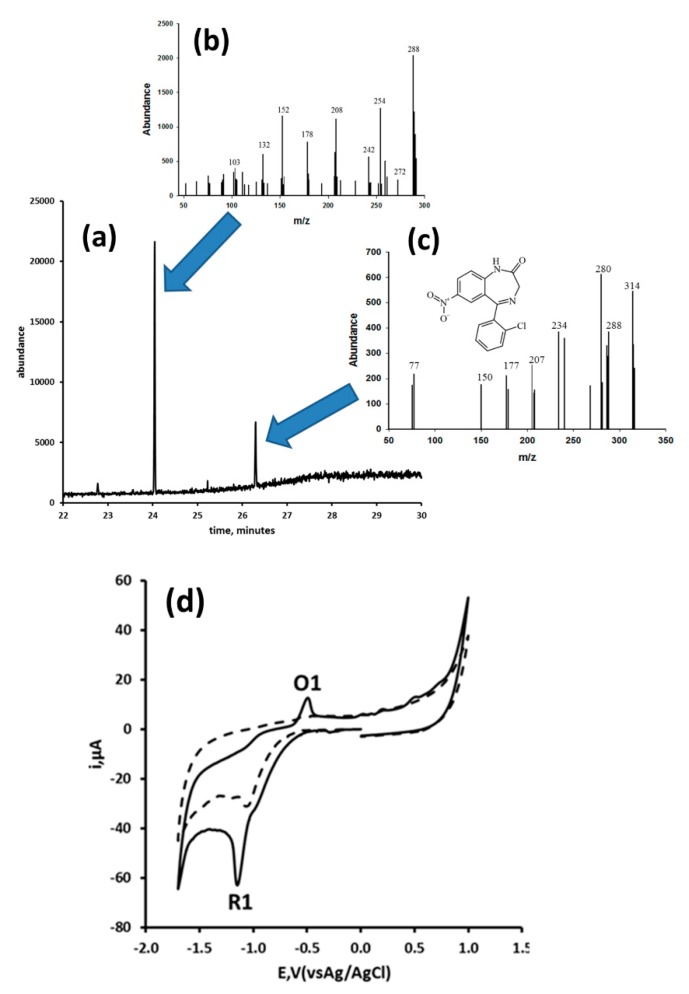
Gas chromatography of clonazepam and possible base hydrolysis product. (**a**) Full scan chromatogram; (**b**) hydrolysis mass spectrum (retention time 24.1 min); (**c**) clonazepam mass spectrum (retention time 26.2 min); and (**d**) cyclic voltammogram obtained in pH 11 phosphate buffer for a 1 mM clonazepam solution [116].

More recently, Honeychurch et al. [115] described the development of a voltammetric method based on a SPCE for the determination of clonazepam in serum and in wine. They first explored the cyclic voltammetric behaviour of clonazepam and the effects of pH and scan rate on the peak current and peak potential of the two reduction peaks and oxidation peaks. The two reduction peaks were recorded on the initial negative going scan, and were considered to be consistent with the 2e^−^, 2H^+^ reduction of the 4,5-azomethine and the 4e^−^, 4H^+^ reduction of the 7-NO_2_ to a hydroxylamine. On the return positive going scan, an oxidation peak was seen, postulated to result from the 2e^−^, 2H^+^ oxidation (O1) of the hydroxylamine to the corresponding nitroso species, similar to that given in Scheme 1 for nitrazepam. Consistent with a number of other benzodiazepines, at pH 11, the solution of clonazepam was found to turn from clear to a yellow colour. Gas chromatography/mass spectrometry (GC/MS) (Figure 1a) was used to investigate the possible reason for this colour formation. Following solvent extraction GC/MS results showed the presence of a compound tentatively identified as the base hydrolysis product of clonazepam (Figure 1b), and a smaller amount of clonazepam (Figure 1c). The resulting cyclic voltammetric peak current (Figure 1d) for this product was found to be directly proportional to scan rate, indicative of adsorption. This adsorption phenomena was exploited to development an adsorptive stripping voltammetric assay for the drug. Using this approach, the experimental conditions required for the differential pulse adsorptive voltammetric measurement of clonazepam in wine and serum samples were optimised. It was reported that this could be performed on sample volumes as small as 100 µL deposited on the SPCE surface. Mean recoveries of 79.53% (%CV = 9.88%) and 88.22% (%CV = 14.1%) were reported for wine fortified with 3.16 µg/mL and serum fortified with 12.6 µg/mL, respectively.

As a result of the association of flunitrazepam (Rohypnol) with drug-facilitated sexual assault, there has been increasing interest in the development of methods capable of determining in beverages at the point of need. Recently [117], Tseliou et al. reported an SPCE-based sensor for the determination of flunitrazepam in untreated, undiluted alcoholic and soft drinks. The sensor is based on modification of the surface of the SPCE via electrical discharge. This was undertaken by inducing multiple electrical sparks from an Fe wire connected to a 1.2 kV transformer across the surface of the SPCE. The SPCE was brought into close proximity (<1 mm) with the Fe wire as cathode (−) and the SPCE as the anode (+). The SPCE was then further modified drop coating a solution of glucose oxidase (GOx) and glucose hydrogel droplets (GluHD) in pH 7 phosphate buffer. Investigations showed the iron spark-treated SPCE to be ca. three times more sensitive towards flunitrazepam compared to the untreated SPCE. Interferences from dissolved oxygen were removed via its reduction in the enzymatic oxidation of GluHD by GOx. This allowed for the voltammetric determination of the reduction of the nitro group of flunitrazepam to be successfully measured unperturbed by the potentially interfering oxygen reduction processes. The detection of flunitrazepam in fortified and unfortified samples of Pepsi Cola^®^, vodka, whisky, tequila, gin, and rum were investigated and the sensor was reported to hold promise as an effective method for the determination of flunitrazepam in such samples.

Garcia-Gutierrez and Lledo-Fernandez [118] utilised an Hg-free SPCE for the voltammetric determination of flunitrazepam (Rohypnol) and 7-amino flunitrazepam in oral fluid, urine, and alcoholic beverages. Cyclic voltammetric investigations demonstrated that flunitrazepam exhibited similar voltammetry behaviour to that shown to nitrazepam described above. However, the authors reported an alternative voltammetric mechanism. Here, the 7-nitro of flunitrazepam was reported to be reduced via 6e^−^, 6H^+^ to the corresponding 7-amino group (Equation (1)). This was oxidised on the return positive going scan to the hydroxylamine at −0.2 V, forming a quasi-reversible pair (Equation (2)). Cyclic voltammetric peak current responses obtained in a pH 6 phosphate buffer solution showed a linear relationship for the oxidation peak obtained at −0.2 V over the concentration range 1.0 to 100 µg/mL.

R-NO_2_ + 6e^−^ + 6H^+^ → R-NH_2_ + 2H_2_O (1)

R-NH_2_ + H_2_O ↔ R-N(H)OH + 2e^−^ + 2H^+^ (2)

The validity of the proposed cyclic voltammetric mechanism was further investigated by undertaking cyclic voltammetric studies on 7-amino-flunitrazepam. The same quasi-reversible pair was obtained, concluded to demonstrate the formation of 7-amino-flunitrazepam to be responsible for the reversible redox pair. The possibility of determining flunitrazepam by cyclic voltammetry in oral fluid, urine, and alcoholic beverages was investigated. The lowest concentration of flunitrazepam that could be detected using cyclic voltammetry was found to correspond to ~30 μg/mL. Chronoamperometry was explored to determine lower levels of flunitrazepam. Using the chronoamperometry response obtained at −0.1 V after 40 s, a linear response was observed over the range 35 to 1100 ng/mL, with a corresponding detection limit of 30 ng/mL (based on 3 σ).

## 5. Ion Selective Electrodes

Ion-selective electrodes offer a number of advantages, including cost and simplicity. Only a small number of reports have been focused on their application for the determination of benzodiazepines, as summarised in Table 3. Salem et al. [135,137] proposed an ion-selective electrode for the determination of diazepam, bromazepam, and clonazepam in tablets and in biological fluids. The electrodes were based on poly(vinyl chloride) or poly(esterurethane)s membranes, doped with drug–tetraphenyl borate or drug–phosphotungstic acid ion pair complexes as electroactive materials.

Al Attas et al. [136] developed an ion-selective electrode for the determination of bromazepam based on the bromazepam–phosphotungestiate ion-association complex as an electroactive material in poly(vinyl chloride) membrane plasticised with o-nitrophenyloctyl ether and dioctyl sebacate. In aqueous solution of pH 3, the sensor was reported to be stable for 4 weeks, giving reproducible potential and linear responses.

## 6. Conclusions

The benzodiazepine class of drugs have been used pharmaceutically since the early 1960s, and is still commonly used medically for a wide number of conditions. Reports have shown that their use in criminal activity and their contamination of the environment have become now more commonly reported problems. Consequently, there is a pressing need for techniques capable of routine monitoring of trace concentrations in a variety of samples. Benzodiazepines are characterised by a relatively easily electrochemically reducible azomethine group, with a number also characterised by groups such as nitro, hydroxyl, and *N*-oxide, making them readily detectable by commonly utilised electrochemical methods and at a range of different electrode materials. A large proportion of the earlier reported research has focused on the use of Hg-based electrodes, characterised by low detection limits in both biological and pharmaceutical samples, requiring little sample preparation. However, in the proceeding five years, reports utilising these electrodes have greatly decreased, probably as a result of changing legislation and attitudes to the toxic effects of this element; nevertheless, methods based on Hg electrodes are still reported.

Applications for the liquid chromatographic electrochemical determination of 1,4-benzodiazeapines have utilised both reductive and, more recently, dual electrode modes of detection. Both approaches have been shown to be selective, sensitive, and capable of determining benzodiazepines in complex samples, such as serum and forensic samples. However, the reductive mode has been shown to suffer from common interferences, such as oxygen.

It is believe that in the future, new analytical developments will continue to be made for other areas other than medical and pharmaceutical analysis, such as in forensic and environmental analysis and has already been shown for the simulation of drug metabolism. The development of new benzodiazepines and applications will also drive the further development of electrochemical assays for this important class of drugs.

## Figures and Tables

**Table 1:** Pre-2006 polarographic and voltammetric methods for the determination of benzodiazepines at mercury-based electrodes.

Benzodiazepine	Electrode Material	Linear Range (M)	Detection Limit (M)	Electrolyte	Waveform and Technique	Sample (s)	Reference
7-acetamido-nitrazepam, dimethyl-diazepam, chlordiazepoxide lactam, 7-amino-diazepam	Dropping mercury electrode	-	-	Britton-Robinson buffer, 0.04 M, plus 0.1 M NaOH to obtain desired pH	DPP	Mechanistic study	[43]
Bromazepam, clonazepam, diazepam, flurazepam, midazolam, and medazepam	Hanging mercury drop electrode	Clonazepam 1 × 10^−7^ to 1 × 10^−5^, bromazepam 1 × 10^−7^ to 1 × 10^−5^, midazolam 1 × 10^−7^ to 1 × 10^−5^, diazepam 1 × 10^−7^ to 1 × 10^−5^, SWCSV clonazepam 1 × 10^−8^ to 8 × 10^−8^¸ bromazepam 1 × 10^−8^ to 8 × 10^−8^, midazolam 1 × 10^−8^ to 8 × 10^−8^, diazepam 1 × 10^−8^ to 8 × 10^–8^, medazepam 1 × 10^−8^ to 8 × 10^−8^	SWV, clonazepam 1.3 × 10^−8^, bromazepam 1.2 × 10^−8^, midazolam 1.6 × 10^−8^, diazepam 1.0 × 10^−8^, SWCSV clonazepam 2.9 × 10^−9^, bromazepam 2.6 × 10^−9^, midazolam 2.0 × 10^−9^, diazepam 2.0 × 10^−9^, medazepam 1.3 × 10^−9^, flurazepam 2.5 × 10^−9^	0.10 M KNO_3_, and Britton–Robinson pH 6.0 buffer	SWV and SWCSV	Tablets	[44]
Diazepam, oxazepam nitrazepam, and flurazepam	Dropping mercury electrode	2 × 10^−7^ to 10 × 10^−7^	2 × 10^−7^	0.2 M acetate pH 4.8 buffer	DPP, DC polarography	Rabbit urine	[45]
Midazolam	Static mercury drop electrode	3.6–36 µM	100 pM	Acetate pH ca. 4.8 buffer	SWAdSV, NPV	-	[46]
Midazolam	Static mercury drop electrode	-	-	0.1 M acetate pH 4.8 buffer	NPV	Mechanistic study	[47]
Midazolam	Hanging mercury drop electrode	1.9 × 10^−6^ to 10^−9^		Britton-Robinson pH 5.00 buffer	AdSV	Pharmaceutical formulations	[48]
Midazolam and metabolites (α-hydroxy-midazolam, 3-hydroxy-midazolam and α,3-hydroxy-midazolam)	Static mercury drop electrode	10^−4^ to 10^−7^	6 × 10^−8^	Sulphuric acid 1.0 M	DPP	-	[49]
Camazepam and bromazepam	Hanging Hg drop electrode	Camazepam 1 × 10^−9^ to 9 × 10^−9^, bromazepam 1 × 10^−8^ × 10^−8^, 270 s at −0.60 V	Camazepam 20 ng/mL and bromazepam 200 ng/mL		DPAdSV	Human serum after isolation with Sep-Pak C_I8_ cartridges	[50]
Flurazepam and metabolites	Dropping mercury electrode	Three orders of magnitude	5 × 10^−8^	Formate pH 4 buffer	DPP	15 mg and 30 mg capsules	[51]
Loprazolam mesilate	Static mercury drop electrode	2.5 × 10^−10^ to 8.0 × 10^−9^	2.5 × 10^−10^	0.04 M ammonium chloride pH 4.0	SWAdCSV	-	[52]
Loprazolam mesilate	Dropping mercury electrode and a static mercury electrode	-	-		DC, AC, normal and inverse pulse polarography, CV, and controlled potential coulometry	Mechanistic study	[53]
Oxazepam and lorazepam	Dropping and hanging mercury electrodes	1.0 × 10^−5^ to 1.5 × 10^−7^ for oxazepam and 2.5 × 10^−7^ to 2.0 × 10^−7^ for lorazepam	1.25 × 10^−7^ for oxazepam, 1.85 × 10^−7^ for lorazepam	Britton-Robinson buffer	DC and AC polarography, CV, DPP, rotating disk voltammetry	Oxazepam in serepax, serenal tablets, and serenid forete capsule; lorazepam in Larpose, activan, trapex tablets	[54]
Oxazepam, lorazepam, and quinazolinecarboxaldehyde	Dropping and hanging mercury electrodes	10^−5^ to 10^−3^ in 10% DMSO/water, 10^−5^ to 10^−4^ in water	-	0.1 M tetraethyl ammonium perchlorate in 90% DMSO/10% water	polarography	Serenid-D and Ativan tablets	[55]
Oxazepam	Forced drop mercury electrode	1 × 10^−5^ to 1 × 10^−2^	-	0.1 M aqueous tetraethyl ammonium iodide	polarography	10 mg tablets	[56]
Halazepam	Dropping and hanging mercury electrodes	1 × 10^−8^ to 1.6 × 10^−7^	25 ng/mL	0.04 M acetate pH 4.8 buffer	DPAdSV −0.5 V	Human serum and urine	[57]
Bromazepam and *N*-1-Desalkylflurazepam	Dropping mercury electrode	5–500 ng/mL	10–20 ng/mL	1 M phosphate pH 4.0 and pH 7.0 buffers containing 0.005% methoxypolyethylene glycol 550	DPP	Human blood	[58]
Flunitrazepam	Hanging mercury drop electrode	0.6 × 10^−8^ to 3.1 × 10^−7^	9.0 × 10^−10^	0.02 M Britton-Robinson pH 10 buffer	Staircase voltammetry and DPAdSV	Urine, following extraction with diethyl ether	[59]
Flunitrazepam	Dropping Hg electrode	1.5 × 10^−5^ to 2.5 × 10^−7^	-	0.2 M boric acid, 0.05 M citric acid, and 0.1 M trisodium orthophosphate pH 4 buffer, containing 0.2% Triton X-100	DPP	Rohypnol tablets	[60]
Clonazepam	Hanging mercury drop electrode	Up to 550 ng/mL	10 ng/mL	0.025 M carbonate pH 10.0 buffer	DPAdSV	Urine, after extraction	[61]
Nitrazepam	Nafion-coated Hg film glassy carbon electrode	Up to 7 × 10^−6^	-	Acetate pH 4.6 buffer	DPAdSV	-	[62]
Nitrazepam	Dropping mercury electrode	-	0.27 µg/mL	McIlvaine pH 3.1 buffer	Polarography	Tablets: Nitravet, Nirven, Sedamon, Hypnotex, Restorem, and Capsule: Hypnotex	[63]
Nitrazepam	Hanging mercury drop	0.5–80 µg/mL in serum by polarography	0.5 µg/mL	Phosphate pH 6.9 buffer	Polarography, CV, chronopotentiometry, controlled coulometry	Serum (without dilution or pre-treatment)	[64]
Nitrazepam, flunitrazepam, and clonazepam as their 2-amino-5-nitrobenzophenones acid hydrolysis products	Glassy carbon electrode	-	-	Polarography, 0.11 M pyridine, and 0.12 M formic acid pH 4.5, CV formate buffer	CV, polarography	-	[65]
Camazepam, oxazepam, and temazepam	Hanging mercury electrode	Oxazepam 2.8 × 10^−8^ to 4.0 × 10^−7^	Oxazepam 3.6 × 10^–10^	0.008 M Britton–Robinson pH 2.0 buffer	DPAdSV	Human serum albumin	[66]
Temazepam	Hanging mercury electrode	10–100 µg/mL		Acetate pH 4.7 buffer solution containing 10% dimethylfonnamide	Polarography and DPP	Soft gelatine capsule formulations after enzyme dissolution	[67]
Pivazepam	Hanging mercury electrode	10^−7^ to 10^−5^	Urine 15 ng/mL, following diethyl ether extraction	DPAdSV 0.1 M McIlvaine pH 4.0 buffer, 10% methanol, DPP 0.04 M Britton-Robinson pH 5 buffer	DPP, DPAdSV	Pharmaceutical tablet formulations and urine	[68]
Chlordiazepoxide, nitrazepam, clonazepam, flunitrazepam, oxazepam, lorazepam, diazepam, bromazepam, prazepam, flurazepam, -otassium chlorazepate, and medazepam	Dropping mercury electrode	-	-	Britton–Robinson pH 4.0 and pH 12.0 buffers	Polarography	Mechanistic studies, hydrolysis products investigated	[69]
chlorazepate	Dropping Hg electrode	10^−7^ to 3 × 10^−5^	ca. 10^−7^	Britton-Robinson pH 4 buffer	Cathode ray polarography	Urine after adjustment to pH 9 and liquid-liquid extraction with ethyl acetate	[70]
Oxazepam	Dropping Hg electrode	-	-	0.1 M HCl/20% methanol	Polarography	Unsuccessful for the direct determination of pharmaceutical formulation	[71]
Diazepam	Dropping mercury electrode	2 × 10^−6^ to 10^−3^	-	Britton-Robinson pH 1.81 buffer—0% methanol	DCP, CV, coulometry	Mechanism study	[72]
Diazepam	Dropping mercury electrode	Alternating current polarography 17.4–167 µM; DPP 4.8–96 µM	-	2 M acetate pH 4.6 solution	DCP, DPP, and alternating current polarography	10 mg tablets	[73]
Diazepam	Hanging mercury drop electrode	-	1 µg/mL	0.1 M KCl solution in 50% ethanol	DPP	Forensic investigations of Toddy (fermented sap or exudate of date, Palmyra, coconut, sago, etc.)	[29]
Diazepam	Hanging mercury drop electrode	5.6 × 10^−8^ to 8.8 × 10^−6^ and 8.8 × 10^−6^ to 2.0 × 10^−4^	9.6 × 10^−9^	0.20 M pH 4.7 acetate buffer, −2.0 × 10^−2^ M K_2_S_2_O_8_	Polarographic parallel catalytic wave of persulfate	Tablets	[74]
Mexazolam	Hanging mercury drop electrode	Up to 13 µg/mL	80 ng/mL		DPP		[75]
Bentazepam	Hanging mercury drop electrode	1.9–9.4 ng/mL by DPAdSV	2.7 ng/mL in urine by DPAdSV; 3.1 × 10^−9^ M by DPP	DPP in 0.04 M Britton-Robinson pH 9 buffer; DPAdSV in 0.08 M phosphate pH 8.0 buffer	DPP, DPAdSV	Tiadipona tablets by DPP and in urine by DPAdSV	[76]
Chlordiazepoxide	Hanging mercury drop electrode	5 × 10^−9^ to 2 × 10^−7^ in human serum by SWAdCSV	4.4 × 10^−10^ and 6.6 × 10^−10^ for pharmaceutical and human serum, respectively, by SWAdCSV	Britton-Robinson pH 8 buffer	Polarography, CV, controlled potential coulometry and SWAdCSV	Bulk form, Librax tablets and human serum (after protein precipitation with ethanol and centrifugation)	[77]
Chlordiazepoxide	Static mercury drop electrode	-	-	0.1 M acetate buffer	SWAdCSV	Synthetic solutions measured in the presence of chloramphenicol, metronidazole, oxytetracycline, cephalothin, and trimethoprim	[78]
Chlordiazepoxide	Hanging mercury drop electrode	Quasilinear over the ranges: 3.20 × 10^−8^ to 1.60 × 10^−7^; 1.60 × 10^−7^ to 1.44 × 10^−6^; 1.44 × 10^−6^ to 1.44 × 10^−5^	9 × 10^−9^	0.12 M NH_3_/NH_4_Cl pH 10.2/0.016 M K_2_S_2_O_6_	Polarography	-	[79]
Chlordiazepoxide	Hanging mercury drop electrode	0.8 to 11 × 10^−8^	0.9 × 10^−9^	Britton-Robinson pH 6.8 buffer	DPAdCSV	Human serum, after solid phase extraction with Sep-Pak C_18_ cartridge	[80]
Chlordiazepoxide	Dropping Hg electrode	2.5 × 10^−6^ to 10^−3^	2.5 × 10^−6^	0.1 M sulphuric acid	Polarography, CV, coulometry	Alpoxide and Prodixamon tablets, serum	[81]
Chlordiazepoxide and metabolites: *N*-desmethylchlordiazepoxide, and demoxepam	Dropping Hg electrode	0.05–1.0 µg/mL	Chlordiazepoxide and its desmethyl metabolite approximately 0.05 µg/mL for plasma; demoxepam, 0.10 µg/mL	0.1 N sulphuric acid	DPP	Plasma after 30 mg dose extracted with diethyl ether at pH 9 and analytes isolation by thin layer chromatography	[82]
Chlordiazepoxide and diazepam	Dropping Hg electrode	-	-	0.1 M sulphuric acid	Automated polarography	Alpoxide and Vival tablets	[83]
Lorazepam	Hanging mercury drop electrode	0.05–1.15 µg/mL	0.019 µg/mL	Britton-Robinson pH 2 buffer	CAdSV	Urine, plasma, Chemidarou and Zahrav tablets	[84]
Lorazepam	Hanging mercury drop electrode		15 ng/mL in urine	Britton-Robinson pH 2 buffer	DPAdSV	Human urine	[85]
Nimetazepam	Dropping Hg electrode	30–150 µg/mL	30 µg/mL	Britton–Robinson pH 3.8 buffer, 50% methanol	Polarography, 0.0 to −0.7 V	Serum and whole blood after extraction with ethyl acetate/toluene 1:1	[86]
Pinazepam and BrTDO	Hanging mercury drop electrode		Pinazepam 2.8 × 10^−10^, BrTDO 2.2 × 10^−10^	0.04 M Britton-Robinson buffers pH 4 and pH 5	DPAdSV	Human serum	[87]
Pinazepam, camazepam, bromazepam, and BrTDO	Hanging mercury drop electrode	Pinazepam 100–700 ng/mL, camazepam 100–1000 ng/mL, bromazepam 100–1000 ng/mL, and BrTDO 100–1000 ng/mL	Pinazepam 15 ng/mL, camazepam 10 ng/mL, bromazepam 20 ng/mL, and BrTDO 20 ng/mL	Britton–Robinson pH 4 buffer for Pinazepam and pH 5 for bromazepam and BrTDO; Acetate pH 5 buffer for camazepam	DPAdSV	Urine after solid-phase extraction with C_18_ Sep-pak	[88]
Timelotem	Dropping mercury electrode	-	2.5 × 10^−6^	Britton–Robinson buffers containing 10% methanol	-	-	[89]
Tetrazepam, nortetrazepam, and menitrazepam	Hanging and dropping mercury electrodes	5 × 10^−7^ to 5 × 10^−6^ tetrazepam	10^−7^	0.1 M sulphuric acid, containing 1% dimethylacetamide	Polarography and DPP	-	[90]
Clotiazepam	Dropping mercury electrode	6.5 × 10^−^^7^ to 1.10 × 10^−^^5^	-	0.1 M sulphuric acid	Polarography and DPP	Distensan tablets	[91]
Estazolam	Dropping mercury electrode	1 × 10^−^^7^ to 9.0 × 10^−^^6^	5 × 10^−8^	0.1 M NH_3_–NH_4_Cl pH 9.2 buffer	Polarography	-	[92]
Camazepam	Dropping mercury electrode	1.3 × 10^−^^6^ to 1.0 × 10^−^^5^ by DPP	1.7 × 10^−8^ by DPP	0.2 M phosphate pH 6.0 buffer	Current sampled polarography, CV, and DPP	Urine, with and without solid phase extraction (Sep-Pak C_18_) and Albego dragees (sugar-coated) 10 and 20 mg tablets	[93]
Metaclazepam	DC tast, DPP and DPAdSV at dropping mercury, hanging mercury electrodes; CV at hanging mercury electrode; coulometry at mercury pool	1.8 × 10^−^^9^ to 3.2 × 10^−7^ by DPAdSV	4.4 × 10^−10^ by DPAdSV	Polarography: Britton-Robinson buffer; coulometry: 4% ethanol/Britton-Robinson buffer	DC tast and DPP, CV, microcoulometry, and DPAdSV	Human urine, 2 mL diluted with 1 mL of 0.04 M pH 9.2 borate buffer; extracted with 1 mL of diethyl ether Evaporated to dryness and reconstituted in ethanol; diluted with 25 mL 0.04 M Britton-Robinson buffer pH 9.1	[94]
Triazolam and clotiazepam	Hanging mercury drop electrode	Up to 2.2 × 10^−7^ for both triazolam and clotiazepam	6 × 10^−10^ for triazolam and clotiazepam with accumulation times of 4 and 10 min by DPAdSV	10^−2^ M carbonate pH 10 buffer	DPAdSV	-	[95]
Diazepam and nitrazepam	Hanging mercury drop electrode	1 × 10^−9^ to 1 × 10^−6^	-	Acetate pH 4.6 buffer	DPAdSV	-	[96]

**Table 2:** Pre-2006 voltammetric methods for the determination of benzodiazepines at carbon-based electrodes.

Benzodiazepine	Electrode Material	Linear Range (M)	Detection Limit (M)	Electrolyte	Waveform and Technique	Sample (s)	Reference
Diazepam, temazepam, and oxazepam	Modified carbon-paste electrodes	Diazepam 0.025–3.0 µg/mL, Temazepam 0.025–0.8 µg/mL, oxazepam 0.025–1.0 µg/mL	Diazepam 0.021 µg/mL, temazepam 0.021 µg/mL, oxazepam 0.012 µg/mL	Britton-Robinson buffer 0.1 M	DPV	Plasma and urine, following SPE	[119]
Loprazolam	Carbon-paste electrode		1.5 × 10^−7^	Britton-Robinson pH 6.3 buffer	DPV	Somnovit (1 mg) tablets	[120]
Flunitrazepam	Bentonite-modified carbon paste	0.2–4.0 µg/mL	0.04 µg/mL	Accumulation media: pH 3.8, adjusted with either nitric acid or potassium hydroxide; measurement media: 0.5 M potassium nitrate pH 3.8	DPV, adsorption at open circuit	Serum and urine	[121]
Olanzapine	Glassy carbon electrode	6.2–50.0 µg/mL	3 µg/mL	0.25 M phosphate pH 2.5 buffer	LSV	Ziprexa tablets	[122]
Bentazepam	C_18_(Bonda pack, 50% *w*/*w*)-modified carbon-paste electrode	0.5–2 µg/mL	0.5 µg/mL	Accumulation media: 0.04 M Britton-Robinson pH 6 buffer; measurement: 0.3 M phosphate pH 2 buffer	DPAdSV, open circuit accumulation, measurement DPV scan 0 to +1.3 V	Human urine	[123]
Olanzapine	Glassy carbon electrode	DPV and OSWV 7.00 × 10^−5^ to 1.00 × 10^−3^	DPV 6.79 × 10^−6^, OSWV 6.91 × 10^−6^	Britton-Robinson pH 3.24 buffer	CV, DPV, and OSWV	10 mg Zyprexa tablets	[124]
Nitrazepam and flunitrazepam	Glassy carbon electrode	5.6–28.1 pg/mL and 6.3–31.3 pg/mL for nitrazepam and flunitrazepam, respectively	1.8 µg/mL and 1.3 µg/mL for nitrazepam and flunitrazepam, respectively	0.1 M phosphate pH 7.0 buffer, containing 10% v/v methanol	Flow injection analysis, 4.0 mL/min, −0.9 V	Tablet formulations of nitrazepam and flunitrazepam	[125]
Clonazepam	Glassy carbon electrode	6.3–31.5 pg/mL	3.3 µg/mL	0.1 M phosphate buffer solution (pH 7.0) containing 10% *v*/*v* methanol	Flow injection analysis, 4.0 mL/min, −0.9 V	Tablet formulations and human urine	[126]
Diazepam	Glassy carbon electrode	1 × 10^−3^ to 1 × 10^−4^	-	HCl–KCl pH 1 buffer containing 10% ethanol	CV	Tablets, capsules, injection, and syrup formulations	[127]
Diazepam	Glassy carbon electrode	0.158–9.48 µM	-	Solution of 0.1 M KCl and 0.01 M pH 4.0 potassium biphthalate buffer	Pulsed rotation voltammetry	-	[128]
Chlordiazepoxide, medazepam, nitrazepam, amino-nitrazepam, lorazepam, and flurazepam	Glassy carbon electrode	-	-	Britton–Robinson pH 4 buffer, containing 10% methanol	Differential pulse flow injection analysis +1.2 V (no oxidation signal seen for nitrazepam)	Tablets	[129]
Tifluadom	C_18_ µBondapak-modified carbon-paste electrode	2.2 × 10^−7^ to 4.5 × 10^−6^	1.3 × 10^−6^ in urine, 1.3 × 10^−7^ in buffer	Britton-Robinson pH 6 buffer	CV	Urine	[130]
Alprazolam	Glassy carbon electrode and static Hg drop electrode	2.5 × 10^−7^ to 3.25 × 10^−5^	-	Britton-Robinson pH 9 buffer	Differential pulse polarography	-	[131]
Flurazepam, oxazepam, medazepam, lorazepam, flunitrazepam, prazepam, potassium chlorazepate, nitrazepam, and amino-nitrazepam	Wall-jet glassy carbon electrode	-	-	Britton-Robinson pH 4 buffer, containing 10% methanol	Differential pulse flow injection analysis +1.2 V	Tablets	[132]
Flurazepam, chlordiazepoxide, oxazepam, lorazepam, potassium chlorazepate, nitrazepam, flunitrazepam, prazepam, diazepamm and medazepam, and some of their metabolites	Wall-jet glassy carbon electrode	5 × 10^−5^ to 2 × 10^−4^	-	0.01 M sodium hydroxide	Differential pulse flow injection analysis +1.2 V	-	[133]
Quetiapine fumarate and olanzapine	Glassy carbon electrode	Quetiapine 3 × 10^−8^ to 4 × 10^−6^, and quetiapine fumarate 2 × 10^−8^ to 5 × 10^−6^	1 × 10^−8^ for both	Britton-Robinson pH 2 buffer	Differential pulse voltammetry	Tablets (Zyprexa and Seroquel), urine, and serum	[134]

Note: Cathodic adsorptive stripping voltammetry (CAdSV), differential pulse adsorptive stripping voltammetry (DPAdSV), differential pulse polarography (DPP), square wave adsorptive stripping voltammetry (SWAdSV), normal pulse voltammetry (NPV), linear sweep voltammetry (LSV), Osteryoung square wave voltammetry (OSWV), cyclic voltammetry (CV), and linear sweep voltammetry (LSV).

**Table 3:** Ion-selective electrodes for the determination of benzodiazepines.

Benzodiazepine	Electrode Material	Linear Range (M)	Detection Limit (M)	Electrolyte		Sample (s)	Reference
Diazepam, bromazepam, and clonazepam	PVC-based ion-selective electrodes	2.5 × 10^−6^ to 10^−4^, 5 × 10^−6^ to 10^−4^, and 2.5 × 10^−4^ to 10^−6^ for diazepam, bromazepam, and clonazepam, respectively	2, 0.307, and 0.9 µM, for diazepam, bromazepam, and clonazepam, respectively	Potentiometric, 10^−4^ M HCl	Ion-selective electrodes	Urine, Vallium, Rivotril, and Calmepam tablets	[135]
Bromazepam	Bromazepam-phosphotungestiate ion association complex in poly(vinyl chloride) membrane	1 × 10^−2^ to 1 × 10^−4^	3 × 10^−5^	Potentiometric	Ion-selective electrodes	Lextonil, Neoopt, and Calmepam tablets	[136]
Diazepam, bromazepam, and clonazepam	PVC solid contact ion-selective electrodes	0.29–71.20, 0.79–31.62, and 0.32–31.57 µg/mL for diazepam, bromazepam, and clonazepam, respectively	0.71, 0.79, and 0.79 µg/mL for diazepam, bromazepam, and clonazepam, respectively	Potentiometric, 10^−4^ M HCL	Ion-selective electrodes	Vallium, Rivotril, and Calmepam tablets	[137]

## Appendix A

Structures of benzodiazepines and related compounds studied in this review.
